# Case Report: Clinical application of immunotherapy-based combination regimen in primary osteosarcoma of the uterus

**DOI:** 10.3389/fonc.2023.1198765

**Published:** 2023-07-04

**Authors:** Jing Yang, Xiaowen Chen, Xiaofang Li, Wenci Liu, Sihai Liao, Yuzhou Wang, Yufang Zuo

**Affiliations:** ^1^ Department of Gynecological Oncology, Cancer Center, Affiliated Hospital of Guangdong Medical University, Zhanjiang, China; ^2^ Department of Pathology, Affiliated Hospital of Guangdong Medical University, Zhanjiang, China; ^3^ Department of Imaging, Affiliated Hospital of Guangdong Medical University, Zhanjiang, China

**Keywords:** sarcomas, osteosarcoma, immunotherapy, targeted therapy, chemotherapy

## Abstract

Primary osteosarcoma of the uterus is an extremely rare pure heterologous sarcoma of the uterus. The relevant available information is limited to case reports. To date, only 31 cases of this type of cancer have been reported. Here, we report the first clinical experience with the administration of an immunotherapy-based combination regimen for multiple metastatic primary osteosarcomas of the uterus. The patient had undergone multiple treatments prior to this regimen, but her condition continued to progress. However, after 3 cycles of immunotherapy combined with targeted therapy and chemotherapy, a review showed that the disease was stable and even in partial remission. The patient has a good quality of life, and long-term survival is expected.

## Introduction

Uterine sarcomas are unusual, accounting for less than 1% of all gynecologic tumors and 3-7% of all uterine tumors. Uterine sarcomas can be classified as homologous (composed of intrinsic uterine components) or heterologous (composed of foreign components such as cartilage and bone). Most uterine sarcomas are homologous, and heterologous uterine osteosarcomas are extremely rare (1). The majority of heterologous uterine tumors are classified as malignant mixed mullerian tumors (MMMTs). Heterologous MMMTs include carcinomas, heterologous sarcomas, and usually homologous mesenchymal components, such as those in leiomyosarcomas or stromal sarcomas. Most uterine sarcomas have been identified as rhabdomyosarcomas or chondrosarcomas, and heterogenous sarcomas have rarely been reported to produce bone and osteophytes (2). In general, the clinical course of osteosarcoma of the uterus is short, probably because it is difficult to detect a pelvic mass until symptoms appear. In addition, osteosarcoma of the uterus has an unusually malignant nature compared to osteosarcomas of other sites (3). The average age of the previously reported patients was 64 years. The majority of patients were perimenopausal or postmenopausal at the time of diagnosis (4). Due to the rarity of primary uterine osteosarcoma, there are currently no effective treatment options for this disease. In recent years, with the rise of immunotherapy and targeted therapy, we have tried to use immunotherapy combined with targeted therapy and chemotherapy to treat this disease. Herein, we report a case of primary uterine osteosarcoma and review the available literature.

## Case report

A 60-year-old female patient presented to a local hospital in February 2021 with irregular vaginal bleeding. At the first visit, vaginal ultrasound showed a heterogeneous hypoechoic mass in the pelvic cavity, with a size of 9.0×7.4 cm. The results of other tests were unknown. An extensive total hysterectomy was subsequently performed.

The postoperative pathological diagnosis was a malignant mesenchymal tumor of the uterus, and the morphology was consistent with a malignant giant cell tumor, accompanied by a large amount of bleeding and necrosis. There was no tumor cell involvement in the bilateral adnexa. No metastasis was found in the lymph nodes examined.

Initial staining showed that the tumor was positive for EMA, CylinD1, Vim, SMA, and CD10, while it was negative for PAX8, ER, PR and S100. Keratin staining did not support the diagnosis of carcinosarcoma, which exhibits neither cartilage formation nor cancerous components. Ultimately, the pathological diagnosis was consistent with high-grade uterine sarcoma with heterologous differentiation (osteosarcoma).

According to the National Comprehensive Cancer Network (NCCN) guidelines for uterine sarcoma, the patient was treated with epirubicin combined with ifosfamide. After 2 cycles of chemotherapy, abdominal magnetic resonance imaging (MRI) showed that the pelvic lesions were smaller than before. Subsequently, chemotherapy was continued for 4 cycles according to the original regimen. A total dosage of 6000 rads of volumetric intensity-modulated radiation therapy was delivered to the pelvic region. Two months later, the patient found a palpable mass of approximately 6.0×6.0 cm in the surgical scar in the lower abdominal wall. Positron emission tomography/computed tomography (PET/CT) showed multiple metastases in the anterior abdominal wall incision, omentum, pelvic mesentery and lungs. After tumor progression, the patient was treated with gemcitabine and docetaxel combined with bevacizumab. After 2 cycles of treatment, abdominal MRI showed that the original mass was significantly larger than before, at approximately 9.7×9.0 cm in size. At that time, the patient was advised to undergo palliative tumor reduction surgery. One month later, preoperative examination was performed, and abdominal CT showed liver metastasis. Considering the risk of surgery and complications, the patient refused to undergo surgical treatment.

**Figure 1:**
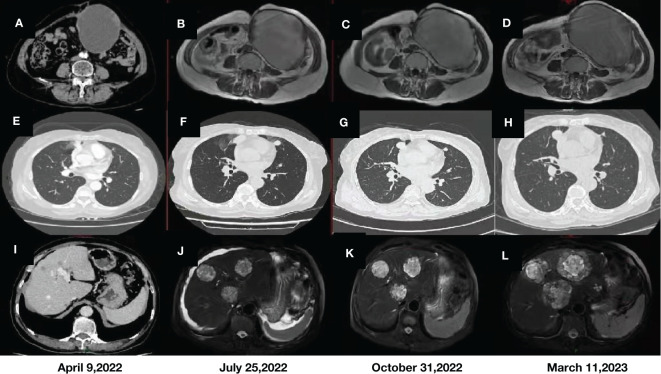
CT and MRI images of the anterior abdominal wall mass **(A, B)** and liver metastases **(I, J)** before immunotherapy. MRI images of the anterior abdominal wall mass **(C, D)** and liver metastases **(K, L)** after immunotherapy. Comparison of CT images of metastatic tumors in both lungs before **(E, F)** and after immunotherapy **(G, H)**.

For further treatment, the patient visited our hospital. Abdominal CT showed large metastases in the anterior abdominal wall and multiple metastases in the liver (**Figures 1A, I**). Chest CT showed multiple metastatic tumors in both lungs (**Figure 1E**). Treatment with tislelizumab and anlotinib was then considered. After 3 cycles of treatment, chest CT showed that the metastatic tumors in both lungs had increased in size, and disease progression was considered. Therefore, paclitaxel injection was added to the original treatment regimen. After 2 cycles of treatment, chest CT and abdominal MRI showed that the metastatic tumors in both lungs, anterior abdominal wall mass and liver metastases were larger than before (**Figures 1B, F, J**). As the disease progressed, the patient gradually developed abdominal pain and bloating.

Given the advantages of current immunotherapy, we tested the patient for programmed cell death-1 (PD-1) and programmed cell death-ligand 1 (PD-L1), but the results were negative (tumor cell (TC)<1%). Eventually, however, we changed the treatment to tislelizumab, liposomal doxorubicin and lenvatinib. After three cycles of treatment, reexamination by abdominal MRI showed no significant changes in the anterior abdominal wall mass and liver metastases (**Figures 1C, K**), but chest CT showed that multiple metastases in both lungs were smaller than before (**Figure 1G**). In addition, the patient’s abdominal pain and bloating symptoms were significantly less than before. Reexamination after 3 cycles of treatment indicated that some of the lung lesions were smaller and some were larger than before (**Figure 1H**), and the anterior abdominal wall masses were the same as before (**Figure 1D**), but the liver metastases were significantly larger (**Figure 1L**). On March 20, 2023, transhepatic arterial chemoembolization and puncture biopsy of the liver mass were performed. The pathological findings after puncture biopsy showed necrotic tissue in some areas, a few heterotrophic cells in some tissues, and pathological mitosis, which were considered to indicate a malignant tumor. Immunohistochemical and genetic analyses were not available because of the small amount of tissue collected. Although the patient’s condition progressed with the previous treatment regimen, her disease was moderately controlled through immunotherapy combined with chemotherapy and targeted therapy, and her survival was expected to be prolonged.

## Discussion

Primary osteosarcoma of the uterus is distinctly rare, and the detailed mechanism of its carcinogenic process remains unclear (5). The available literature contains little information on its relative incidence, clinical behavior, and treatment outcomes (4). A review of the available literature (**Table 1**) showed that the previously reported patients ranged in age from 41 to 82 years, and all but 3 patients were perimenopausal or menopausal at the time of diagnosis. The clinical manifestations of most patients were vaginal bleeding and abdominal pain (1–19). In our case, the patient presented with vaginal bleeding in the early stage and mainly abdominal distension in the later stage.

**Table 1:** Primary uterine osteosarcoma literature review.

Author	Age	Symptoms	Extension of tumor, size	Treatment	Prognosis
Stier and Lyman (1936) (4)	53	Lower abdominal pain,12 months	Uterus and omentum,6cm	Subtotal hysterectomy, BSO	Died at 2 months (recurrence and pulmonary metastasis)
Bickel et al. (1956) (4)	73	Vaginal bleeding,3 months	Uterus	Intracavitary radium,TAH	Died at 20 months (osseous metastasis)
Scheffey et al. (1956) (4)	67	Abdominal discomfort,vaginal bleeding,3 months	Uterus with intraoperative rupture, 4 cm	TAH,BSO	Died at 6 months with spread to abdominal wall
Radman and Korman (1960) (4)	60	Vaginal bleeding, 3-4 weeks	Uterus	TAH,BSO	Died soon after discharge
Carleton and Williamson (1961) (4)	82	Vaginal bleeding and discharge, 8 months	Uterus, extending to base of the bladder, metastasizing to lungs (autopsy)	Intracavitary radium	Died at 8 months (lung metastases at autopsy)
Amromin and Gildenhorn (1962) (4)	72	Vaginal bleeding, weight loss	Uterus, peritoneum, small bowel,40cm	Intracavitary radium	Died at 2 months (intraabdominal metastases)
Karpas and Merendino (1964 ) (4)	62	Vaginal bleeding	Uterus, small bowel, bladder,8cm	TAH,BSO	Lost to follow-up
Crum et al. (1980) (2)	41	Vaginal bleeding, 1 month	Uterine cervix, 9cm	TAH, BSO, RT, CHT	Alive at 4 months
Vakiani et al. (1982) (4)	53	Vaginal bleeding	Uterus, 13 cm	TAH, BSO, CHT	Alive at 1 year
Piscioli et al. (1985) (6)	56	Vaginal bleeding	Uterus, 12 cm	TAH, BSO, RT	Died at 37 months (lung metastases)
Jotkowitz and Valentine (1985) (7)	51	Backache, abdominal pain, weight loss	Uterus, pelvis, omentum, peritoneum	None	Died at 20 days
Basolo et al. (1988) (4)	60	Abdominal pain, vaginal bleeding	Uterus	Radical Wertheim hysterectomy	Lost to follow-up
Caputo et al. (1990) (8)	58	Vaginal bleeding	Uterus, urinary bladder, rectum, uterine cervix, vagina (autopsy), 18 cm	None	Died at 2 weeks (regional lymph nodes, liver and lung metastases at autopsy)
De Young et al. (1992) (9)	63	Uterine bleeding	Uterus, 7 cm	TAH, BSO	Died at 20 days after surgery due to myocardial infarction
Emoto et al. (1994) (10)	67	Abdominal pain	Uterus; lung metastases, 16 cm	TAH, BSO	Died at 4 months (local recurrence and distant metastases)
Akiba et al. (1994) (11)	73	NA	Uterus,10cm	TAH, BSO	Alive at 20 months; lung metastases
Hardisson et al. (2001) (4)	41	Vaginal bleeding	Uterus, 8 cm	TAH, BSO, CHT, RT	Alive at 8 months; tumor recurrence
Su et al. (2002) (3)	62	Abdominal pain	Uterus, 20 cm	Biopsy of mass	Died at 4 months (Lung, thyroid, and Peritoneum metastases)
Lin et al. (2002) (12)	67	Lower abdominal pain for a month	Uterus, peritoneum	OMT, CHT	Alive at 6 months
Kostopoulou et al. (2002) (13)	56	abdominal distention and lower abdominal pain	Uterus, the right adnexum and the cecum	TAH, BSO	Died at 6 months
Ribeiro-Silva et al. (2004) (14)	60	abdominal pain, 2 years	Uterus	hysterectomy	NA
Wang et al. (2011) (15)	53	Vaginal bleeding	Uterus, 8 cm	RH,OMT,CHT	Died at 5 months
Kefeli et al. (2012) (15)	53	Vaginal bleeding	Uterus, 19 cm	TAH, BSO	NA
Powell et al. (2014) (16)	60	Vaginal bleeding	Uterus, 12 cm	TAH, BSO	Died at 7 months (local recurrence and lung metastases)
Abraham et al. (2015) (1)	47	Abdominal pain, vaginal bleeding, early satiety, chronic cough	Uterus, 14 cm	TAH, BSO, CHT	Died at 6 months (cardiac and lung metastases)
Tsukasaki et al. (2016) (15)	57	Abdominal pain	Uterus, 12 cm	TAH, BSO, appendectomy,CHT	Alive at 13 months (local recurrence and lung metastases)
Zheng et al. (2019) (5)	74	postmenopausal bleeding, bloating, and weight loss	Uterus	TAH, BSO,CHT, palliative radiation therapy	Died at 7 months with multiple distant metastases
Yang et al. (2020) (17)	50	None	Uterus, 12cm	TAH, BSO, CHT	Died at 8 months (lung and brain metastases)
Effah et al. (2021) (18)	60	postmenopausal bleeding, weight loss	Uterus	TAH, BSO	Died at 14 months after surgery
Effah et al. (2021) (18)	42	lower abdominal pain	Uterus,sigmoid colon, the upper rectum	TAH, BSO,resection of the sigmoid colon and upper rectum with the construction of colostomy, radiotherapy	Died at 4 months after surgery
Ruhotina et al. (2022) (19)	57	Abdominal pain	Uterus, 15cm	TAH, BSO, CHT	Alive at 12 months (Peritoneal metastasis)
This case	60	Vaginal bleeding	Uterus, 9 cm	Extensive total hysterectomy, CHT, RT, antiangiogenic therapy, targeted therapy, immunotherapy	Alive at 25 months (multiple distant metastases)

TAH, total abdominal hysterectomy; BSO, bilateral salpingo-oophorectomy; CHT, chemotherapy; RT, external radiotherapy; RH, radical hysterectomy; OMT, omentectomy; NA, not available/reported.

**Figure 2:**
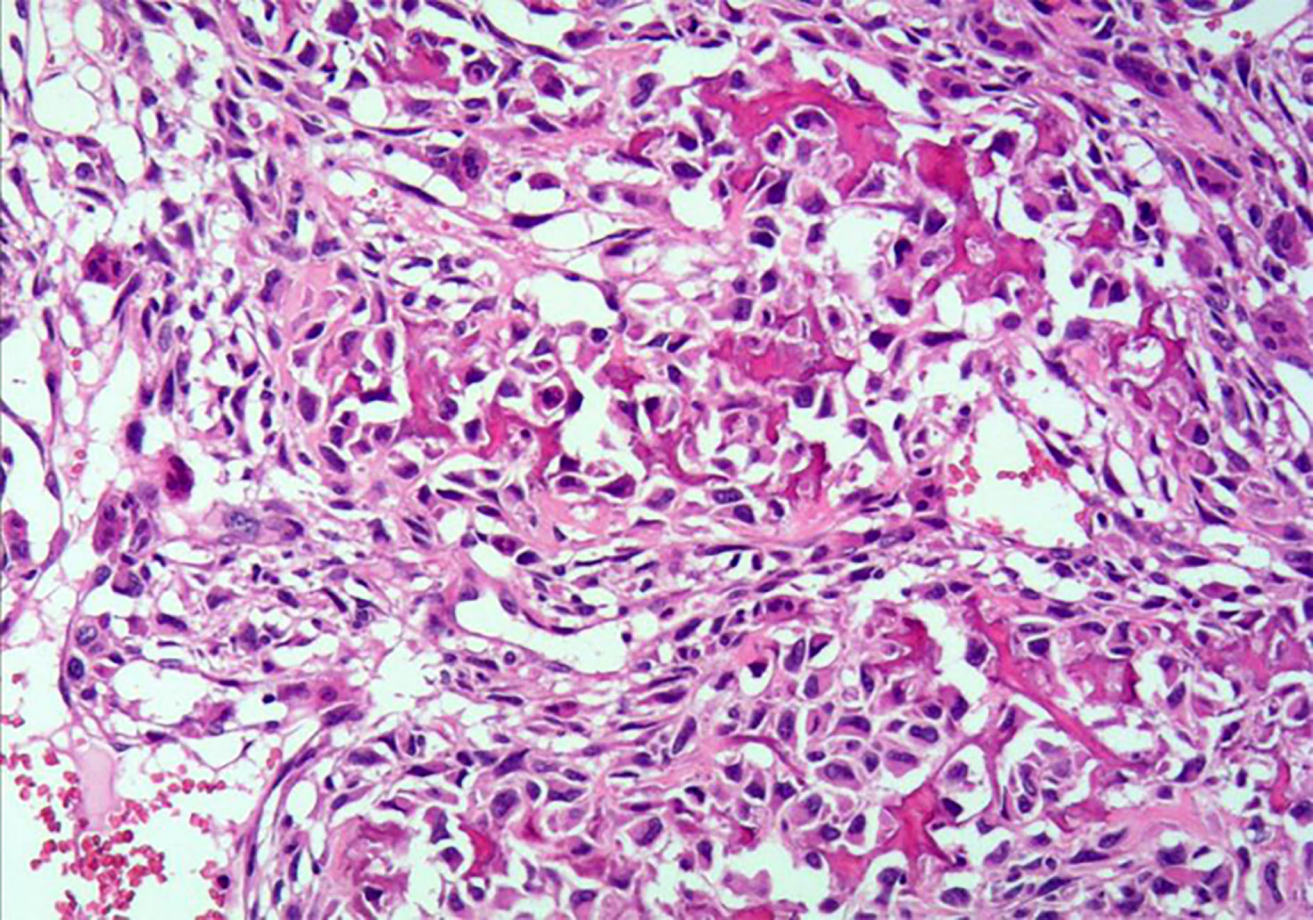
Hematoxylin and eosin staining of primary uterine osteosarcoma tissue sample (under 200× magnification).

Microscopically, uterine malignancy was associated with extensive necrosis, neoplastic osteogenesis in most areas, diffuse distribution of giant cells, local spindle-shaped tumor cells, rich red cytoplasm, severe nuclear atypia, and clear mitosis (**Figure 2**). According to the description published by Piscioli et al., primary uterine osteosarcoma must meet the following three criteria: exclusion of a primary source of bone, presence of neoplastic osteoids, and absence of epithelial components and other specific homologous or heterologous components after confirmation of the presence of sufficient tissue samples (1, 6). Accordingly, the histological findings of this patient fully met the above criteria.

**Figure 3:**
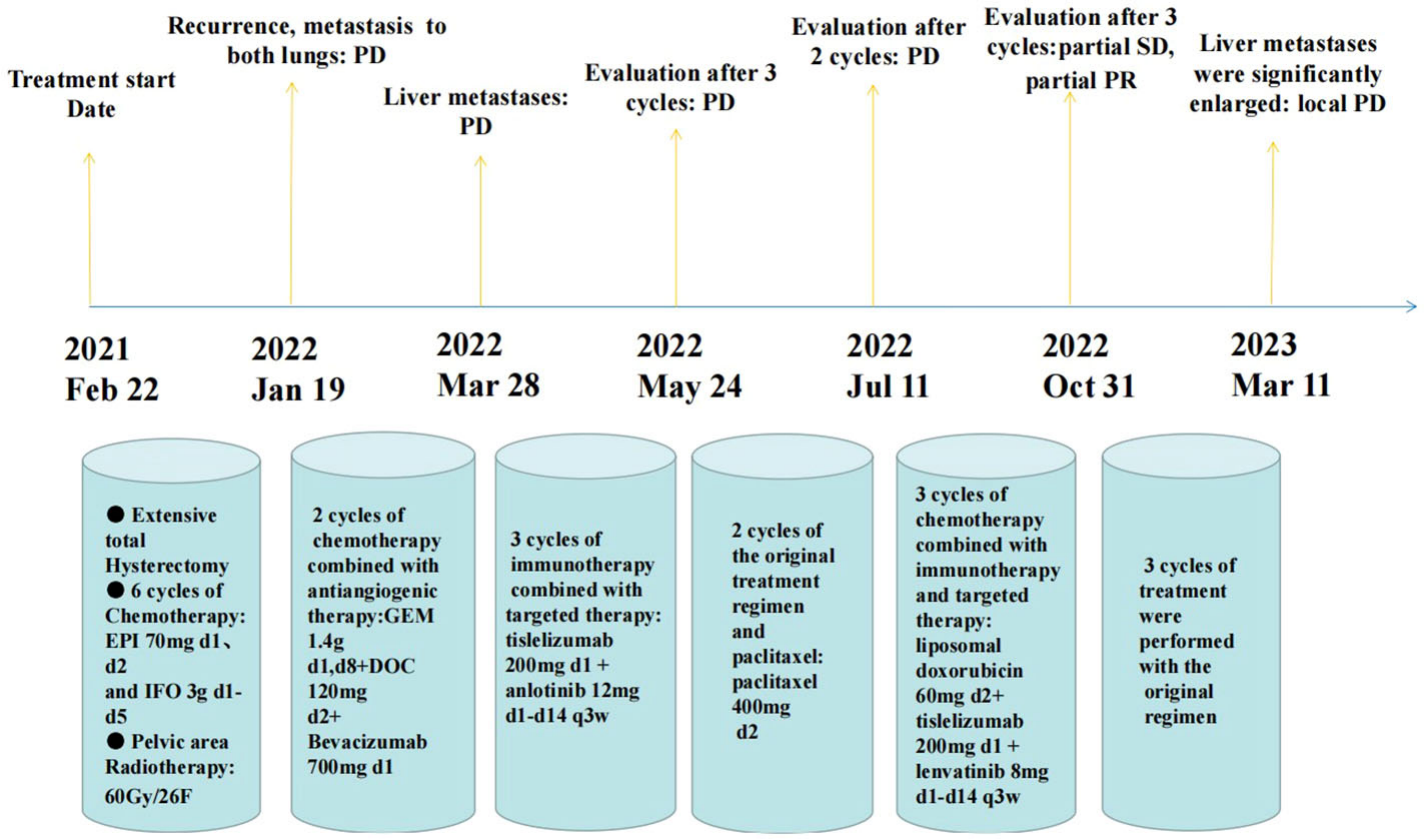
A nearly 25-month treatment history of the patient with primary uterine osteosarcoma. (PD, progressive disease; SD, stable disease; PR, partial remission).

This tumor is highly aggressive with a poor prognosis, and the mean survival time is 8.1 months (15). As shown in the **Table 1**, hysterectomy was performed in 24 patients, and bilateral salpingo-oophorectomy was also performed in 21 of these patients. Seven patients were treated with radiation therapy, including luminal radium therapy and external radiation therapy. Eleven patients received chemotherapy. The table shows that even after a combination of surgery, radiotherapy and chemotherapy, the treatment outcome is still unsatisfactory. Survival ranged from 2 weeks to 20 months, except for one patient who had survived for 37 months. In comparison, our patient has now survived for 25 months, and the survival time is expected to be extended. The results of tracking indicate that this cancer has a high probability of recurrence and distant metastasis, as well as a mortality rate. Regardless of treatment, most patients develop local or pulmonary metastases early after surgery and die within one year of starting treatment (15). Due to the rarity of uterine osteosarcoma, there is currently no standard for treatment. In this patient, previous first- and second-line treatments for uterine sarcoma failed. Her third-line therapy was challenging, and we referred to the NCCN guidelines for soft tissue sarcoma to recommend the application of pembrolizumab in third-line therapy (20). Due to the patient’s financial situation, we chose domestic tislelizumab as an alternative. Moreover, because our hospital only has SP263 antibody to detect the expression of PD-L1, although the result showed TC < 1%, this result may not necessarily reflect the real immune microenvironment of the patient. Sarcomas are cold tumors, and the effect of immunotherapy alone is poor. Existing studies have shown that the immune microenvironment can be changed by using targeted drugs to turn cold tumors into hot tumors (21, 22), and anlotinib is recommended in the domestic guidelines for soft tissue sarcoma (23); thus, we chose tislelizumab in combination with anlotinib. However, after treatment with this regimen, the patient’s disease progressed. We reviewed the NCCN guidelines for endometrial cancer and found that lenvatinib in combination with pembrolizumab has been approved for advanced endometrial cancer with pMMR (24). Therefore, we replaced anlotinib with lenvatinib. Considering the poor effect of tislelizumab in combination with anlotinib in the treatment of this patient, we referred to the NCCN guidelines for uterine sarcoma and added liposomal doxorubicin (25). As a combined result of the above analysis, we chose a regimen of lenvatinib in combination with tislelizumab and liposomal doxorubicin to treat this patient. As a result, the patient’s condition remained stable, with no obvious adverse reactions, and the symptoms of abdominal pain and abdominal distension were relieved. The patient has survived for nearly 25 months since treatment began and is in good condition (**Figure 3**). This is the first clinical experience with using a combination regimen based on immunotherapy to treat this disease. This treatment regimen may serve as a new option for controlling this tumor.

Since this study is a case report, there are not sufficient data to support the efficacy of this treatment regimen. As the incidence of primary uterine osteosarcoma is extremely low, there are few relevant studies, all of which are case reports. There are not enough patients to conduct a large randomized controlled clinical trial, which is a limitation for the future treatment of this tumor. For this rare disease, a global multicenter collaboration is needed to conduct analyses of larger cohorts.

## Data availability statement

The original contributions presented in the study are included in the article/supplementary material. Further inquiries can be directed to the corresponding authors.

## Ethics statement

Written informed consent was obtained from the individual(s) for the publication of any potentially identifiable images or data included in this article.

## Author contributions

Data collection and article writing: JY, XC. Pathological picture analysis: XL. Image analysis: WL. Clinical data analysis: SL, YW. Subject design and article revision: YZ. All authors contributed to the article and approved the submitted version.
